# Supplementary material for: Evaluation of the Effectiveness of Derived Features of AlphaFold2 on Single-Sequence Protein Binding Site Prediction
Source: Biology (Basel). 2022 Oct 3;11(10):1454. doi: 10.3390/biology11101454 (PMC9598995; doi:10.3390/biology11101454)

Table S1. The MaxASA values used in this study. The MaxASA values are measured in Å^2.

Residue	MaxASA	Residue	MaxASA
**Alanine**	129	Leucine	201
**Arginine**	274	Lysine	236
**Asparagine**	195	Methionine	224
**Aspartate**	193	Phenylalanine	240
**Cysteine**	167	Proline	159
**Glutamate**	223	Serine	155
**Glutamine**	225	Threonine	172
**Glycine**	104	Tryptophan	285
**Histidine**	224	Tyrosine	263
**Isoleucine**	197	Valine	174

Figure S1. Visualization of 3D structure predicted by AlphaFold2 of P15309 (colored in yellow) and P15309_V30A (colored in blue), the RMSD between these two structures is 0.050 Å (calculated by PYMOL). The mutation site is colored in red (pointed by an arrow).

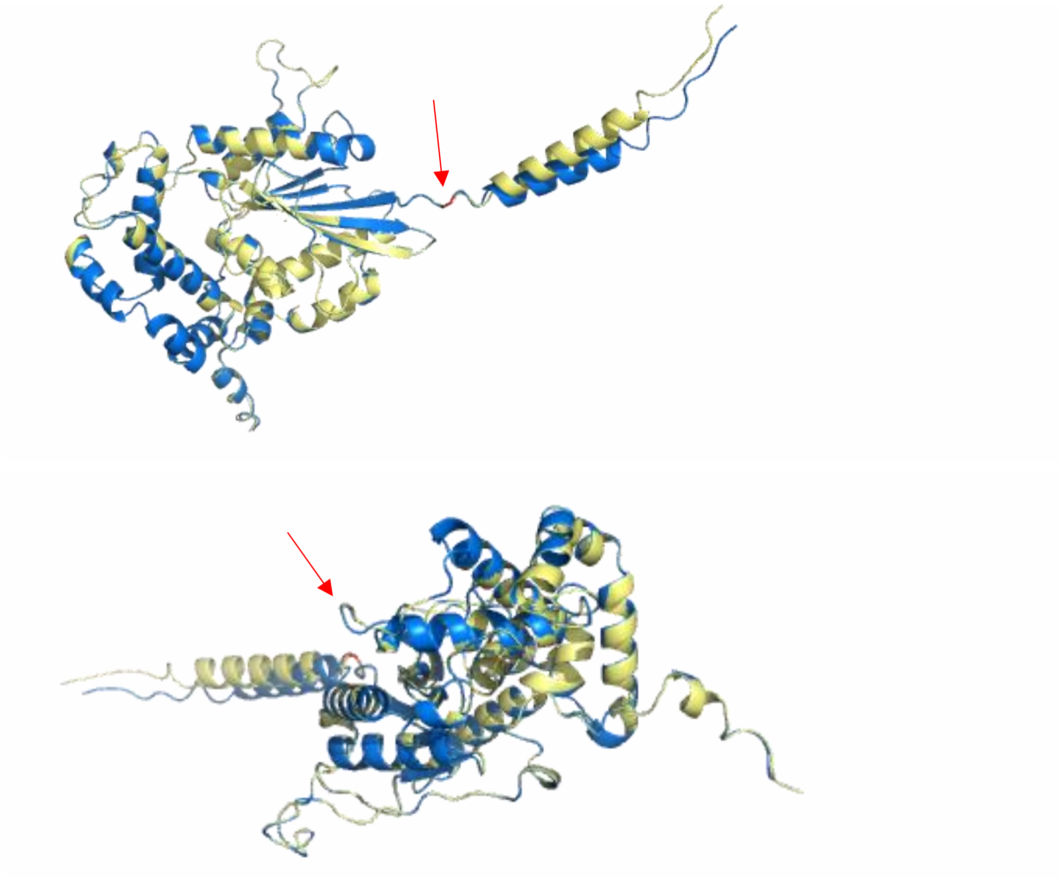

Figure S2. Visualization of 3D structure predicted by AlphaFold2 of O60671 (colored in yellow) and O06071_I30A (colored in blue), the RMSD between these two

structures is 0.441 Å (calculated by PYMOL). The mutation site is colored in red (pointed by an arrow).

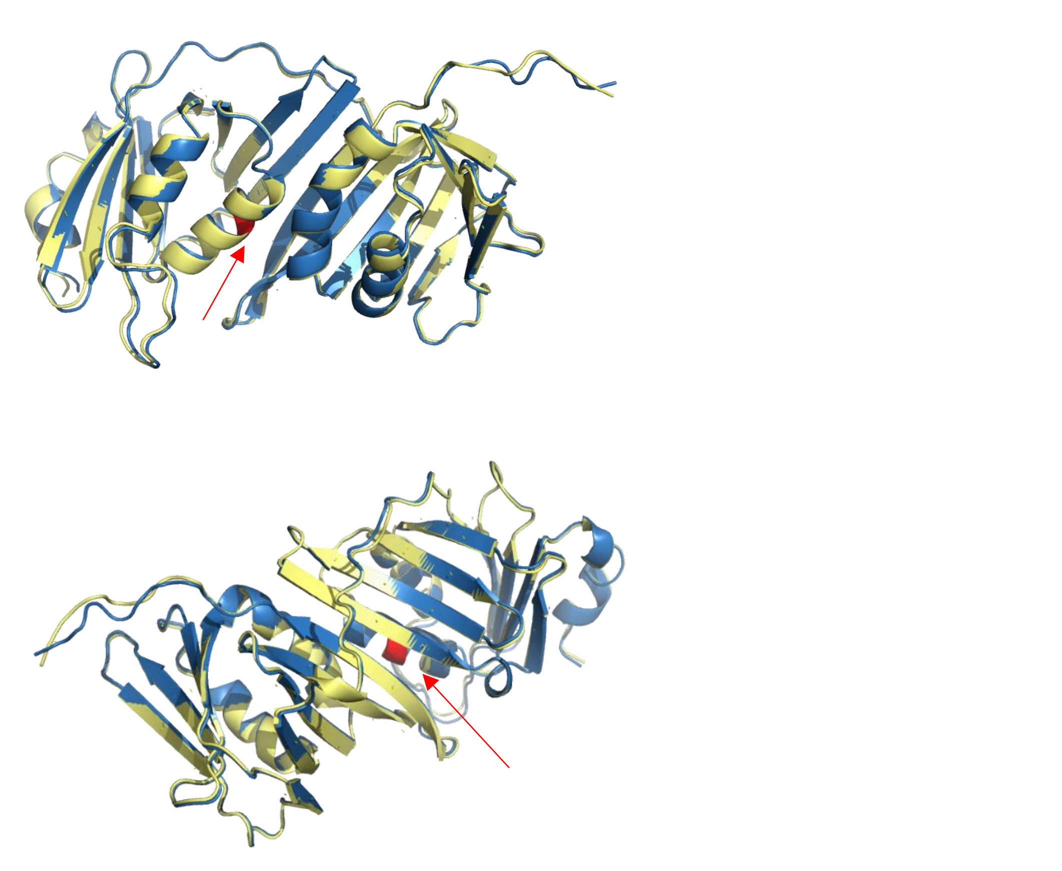

Figure S3. Visualization of 3D structure predicted by AlphaFold2 of Q9UIL1 (colored in yellow) and Q9UIL1_R30A (colored in blue), the RMSD between these two structures is 2.264 Å (calculated by PYMOL). The mutation site is colored in red (pointed by an arrow).

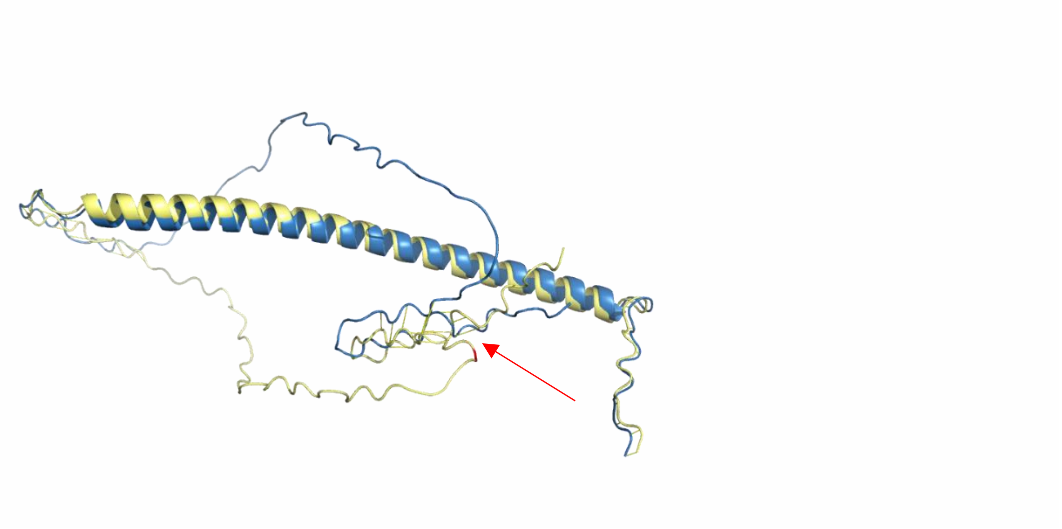

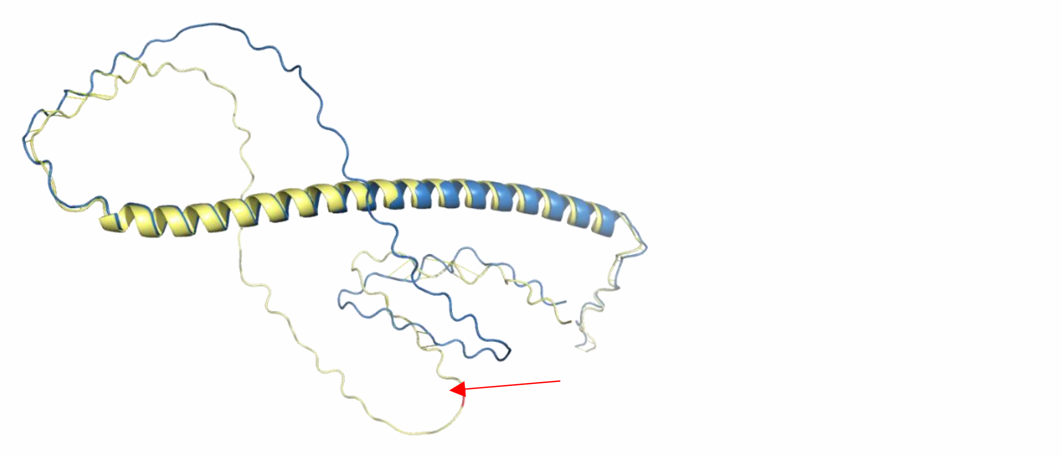

Supplement: Supplementary file 1 [file biology-11-01454-s001.zip › biology-1927656-supplementary.pdf]
